# EZH2 regulates neuroepithelium structure and neuroblast proliferation by repressing p21

**DOI:** 10.1098/rsob.150227

**Published:** 2016-04-20

**Authors:** Naiara Akizu, María Alejandra García, Conchi Estarás, Raquel Fueyo, Carmen Badosa, Xavier de la Cruz, Marian A. Martínez-Balbás

**Affiliations:** 1 Department of Molecular Genomics, Instituto de Biología Molecular de Barcelona (IBMB), Consejo Superior de Investigaciones Científicas (CSIC), Barcelona 08028, Spain; 2 Vall d'Hebron Institute of Research (VHIR), Passeig de la Vall d'Hebron, 119, Barcelona 08035, Spain; 3 Institut Català per la Recerca i Estudis Avançats (ICREA), Barcelona 08018, Spain

**Keywords:** EZH2, neuroblast proliferation, gene silencing, histone methylation, neural development

## Abstract

The function of EZH2 as a transcription repressor is well characterized. However, its role during vertebrate development is still poorly understood, particularly in neurogenesis. Here, we uncover the role of EZH2 in controlling the integrity of the neural tube and allowing proper progenitor proliferation. We demonstrate that knocking down the EZH2 in chick embryo neural tubes unexpectedly disrupts the neuroepithelium (NE) structure, correlating with alteration of the Rho pathway, and reduces neural progenitor proliferation. Moreover, we use transcriptional profiling and functional assays to show that EZH2-mediated repression of p21^WAF1/CIP1^ contributes to both processes. Accordingly, overexpression of cytoplasmic p21^WAF1/CIP1^ induces NE structural alterations and p21^WAF1/CIP1^ suppression rescues proliferation defects and partially compensates for the structural alterations and the Rho activity. Overall, our findings describe a new role of EZH2 in controlling the NE integrity in the neural tube to allow proper progenitor proliferation.

## 1. Introduction

Epigenetic factors are essential for tissue development and cellular homeostasis. The major class of epigenetic regulators in developmental contexts is the Polycomb group (PcG) proteins [1]. PcG proteins control gene expression by keeping many developmental regulators silenced [2]. The hallmark for Polycomb-mediated repression is methylation of lysine 27 of histone H3 (H3K27me3) [3,4], catalysed by Polycomb repressive complex 2 (PRC2) subunits enhancer of zeste homologues 1 and 2 (EZH1/2) [3,5–8]. While EZH1 is predominant in differentiated tissue, EZH2 is highly expressed in proliferating cells, such as embryonic and adult neural progenitors [8–13], and in a wide variety of cancer cells [14,15]. PRC2 is required for proper differentiation of embryonic stem cells (ESCs) and somatic stem cells [8,12]. There, EZH2 controls cell proliferation in part by regulation of the Ink4A/Arf locus [12]. In addition, EZH2 can control cofilin activity and, consequently, the actin cytoskeleton structure that regulates the expression of integrin alpha 2 in colon cancer cells [16]. In addition to its role as an epigenetic factor, EZH2 cooperates with different signals to allow actin reorganization and proliferation [17].

The previous data show that the function of EZH2 as a transcription repressor is well characterized. However, its role during vertebrate development is still poorly understood, particularly in neurogenesis. Several models have been generated to study the role of PRC2 during neural development [18,19]. We know that, at the onset of neurogenesis, conditional deletion of EZH2 in the cerebral cortex shifts the balance between self-renewal and differentiation towards the latter [19]. This leads to a decreased number of neurons at birth. By contrast, it has been found that EZH2 deletion at a later time in neural precursors results in an increased number of neurons [18]. These data indicate that EZH2 has a strict timing-dependent role in neurogenesis, but do not fully clarify the role of PRC2 in early neural development. To explore the potential contribution of PRC2 to event coordination during this stage, we analysed the consequences of EZH2 depletion in chick embryo spinal cord. We found that knocking down EZH2 in neural progenitors disrupts the neuroepithelium (NE) structure and reduces their proliferation capacity. In addition, we observed that *p21^WAF1/CIP1^* repression by EZH2 contributes to both processes. Our data demonstrate that EZH2 is necessary to maintain the NE integrity, allowing proliferation of neuroepithelial cells in the neural tube.

## 2. Results

### 2.1. EZH2 is required for neural progenitor proliferation and survival

**Figure 1.**
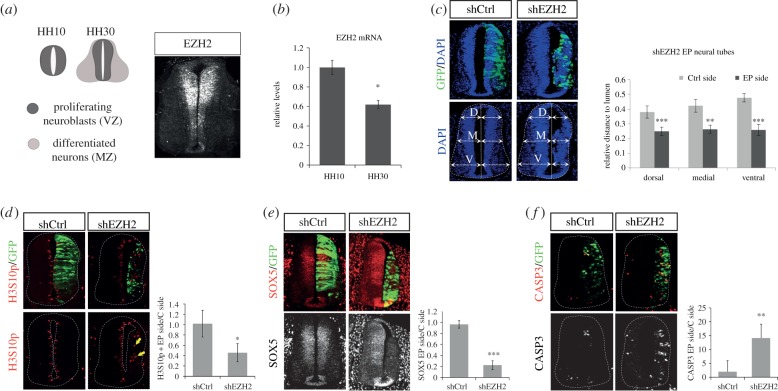
EZH2 depletion leads to small and structurally disorganized neural tubes. (*a*) Diagrams show regions occupied by proliferating neural progenitors (ventricular zone, VZ) and post-mitotic neurons (mantle zone, MZ) in HH10 (only progenitors) and HH30 chick embryo spinal cord (left panel). HH30 wild-type embryo neural tubes were immunostained using anti-EZH2 antibody (right panel). (*b*) Ten HH10 or HH30 embryo neural tubes were dissected out for each replicate. mRNA was extracted and retrotranscribed for qPCR analysis. The graph shows EZH2 mRNA, normalized by GAPDH mRNA levels. Results are means of three independent experiments. Error bars indicate s.d. *p < 0.05. (*c*) HH11–12 embryos were co-electroporated with a mixture of shRNA-EZH2(1) and shRNA-EZH2(2) (shEZH2) or shRNA-control (shCtrl) cloned into pSHIN vector expressing GFP. Transversal sections of electroporated neural tubes as indicated above stained with DAPI 48 h PE. Graphs show the quantification of the size of the control side and shEZH2-electroporated side. To do that, we measured the dorsal, medial and ventral distances to the lumen on each side, relative to the length of the central line of the lumen. Data represent mean of *n* = 10–20 sections (from three to five embryos). Error bars indicate s.d. **p < 0.01; ***p < 0.001. (*d*–*f*) Transversal sections of neural tubes from HH11–12 embryos electroporated *in ovo* with shCtrl or shEZH2 and stained for H3S10p (*d*), Sox5 (*e*) or caspase 3 (*f*) 48 h PE. Graphs show the quantification of the corresponding immunostaining. Data represent mean of *n* = 20–30 sections (from four to six embryos). Error bars indicate s.d. *p < 0.05; **p < 0.01; ***p < 0.001.

*EZH2* is highly expressed in the ventricular zone (VZ, formed by neural progenitors) of the developing chick spinal cord (figure 1*a*). Moreover, we observed that its expression decreased in Hamburger and Hamilton (HH) stage 30 embryos, where the mantle zone (MZ, formed by neurons) is predominant, compared with HH10 embryos (figure 1*a,b*). These observations led us to study whether PRC2 complex is required during early stages of neurogenesis. For this purpose, we knocked down *EZH2* expression in HH 11–12 chick embryo spinal cords, by *in ovo* electroporation of two distinct non-overlapping short hairpin (sh)RNAs (shEZH2(1) or shEZH2(2), see sequences in Material and Methods section) that efficiently reduced EZH2 levels (electronic supplementary material, figure S1*a*), or a control shRNA (shCtrl). Remarkably, *EZH2* knock down resulted in a small and structurally disorganized neural tube (electronic supplementary material, figure S1*b*). As the two independent shRNA-EZH2 sequences gave similar phenotypes, we co-electroporated both shEZH2(1) and shEZH2(2) sequences (that we named shEZH2) in the remaining experiments. As expected, this process efficiently decreased the EZH2 RNA (electronic supplementary material, figure S1*c*) and protein levels (55%) (electronic supplementary material, figure S1*d*) 48 h post-electroporation (PE), and resulted in a reduction of the electroporated side and a disorganized neural tube (figure 1*c*). At an earlier time (24 h PE), lower EZH2 reduction (30%) was observed (data not shown). Thus, we decided to perform the rest of the analysis at 48 h PE.

To further investigate shEZH2 specificity, we performed rescue experiments overexpressing human *EZH2* (rEZH2), which is not targeted by chicken shEZH2. The data in the electronic supplementary material, figure S1*e* show the rescue of both the size and morphological alterations of the neural tube after electroporation of the rEZH2 plasmid, demonstrating the specificity of the shEZH2.

As EZH2 is the catalytic subunit of PRC2, we assessed if EZH2 depletion affected global H3K27me3 levels. Immunostaining assays showed no changes in global H3K27me3 (electronic supplementary material, figure S2*a*), which might be explained by an increase of EZH1 after EZH2 depletion (electronic supplementary material, figure S2*b*). We further analysed the contribution of H3K27me3 mediation in the role of EZH2. We performed rescue experiments overexpressing rEZH2 mutated at the catalytic SET domain that lacks histone methyltransferase (HMT) activity. The results indicated that neither the size nor the morphological alterations of the neural tube were rescued to the same extent as in the EZH2 wild-type (electronic supplementary material, figure S2*c*). These data suggest that H3K27me3 or methylation of an unknown substrate contributes to the described phenotype.

The role of EZH2 in proliferation has been well characterized in ESCs [12,14,20,21]. On this basis, we hypothesized that the reduced size of shEZH2-electroporated neural tubes might reflect an active role of EZH2 in the maintenance of neural progenitor proliferation, following a previous proposal [19]. To confirm this, we electroporated HH11–12 neural tubes with shEZH2 or shCtrl and analysed the effect on neural progenitor proliferation after 48 h. We evaluated the entry of neural progenitors into the S phase of the cell cycle, analysing bromodeoxyuridine (BrdU) incorporation. In the electronic supplementary material, figure S3*a*, we show that shEZH2-electroporated cells (green fluorescent protein (GFP+)) incorporated less BrdU than those electroporated with shCtrl (percentage of BrdU+GFP+/total GFP+ cells: shCtrl 19.4 ± 6.1%, shEZH2 11.5 ± 4.9%; *p* < 0.01). Accordingly, neural tubes electroporated with shEZH2 showed a reduction in H3S10p-positive mitotic cells (ratio of H3S10p+ cells electroporated (EP) side/control side: shCtrl 1.02 ± 0.26, shEZH2 0.46 ± 0.18; *p* < 0.001) (figure 1*d*). Finally, reduction of the neural progenitor marker SOX5 (ratio of SOX5+ cells EP side/control side: shCtrl 0.97 ± 0.07, shEZH2 0.22 ± 0.08; *p* < 0.001) (figure 1*e*) suggests that EZH2 is required for neural progenitor self-renewal in the neural tube.

As a growing number of studies demonstrate that PcG proteins are required at promoters of many neurogenesis-specific genes [22,23], we then decided to explore if inhibition of proliferation upon EZH2 reduction corresponded with a premature differentiation of neuroblasts. We found that neural tubes electroporated *in ovo* with shEZH2 and stained for Tuj1, a neuronal differentiation marker, showed premature differentiation and an ectopic localization at 48 h PE (electronic supplementary material, figure S3*b*). However, we did not observe an increase in the number of differentiated neurons (ratio of Tuj-1+ cells EP side/control side: shCtrl 0.95 ± 0.09, shEZH2 0.90 ± 0.08). Next, we tested the presence of cellular death after EZH2 depletion. By active caspase 3 immunostaining, we observed a higher number of apoptotic cells in the shEZH2-electroporated side of the neural tube than in the shCtrl-electroporated side (ratio of caspase 3+ cells EP side/control side: shCtrl 2 ± 3.95, shEZH2 14.1 ± 5.1; *p* < 0.01; figure 1*f*).

Finally, we showed that shEZH2-electroporated embryos display ectopic mitotic cells far from the apical membrane, their typical location in wild-type embryos (figure 1*d*, indicated by arrows). This result suggests either an incomplete interkinetic nuclear movement or a consequence of the ectopic formation of new apical membrane-like structures (see below).

Taken together, these results indicate than EZH2 is essential to preserve the NE structure of the neural tube and to maintain proliferation and survival of neural progenitor cells.

### 2.2. EZH2 preserves NE structure

**Figure 2.**
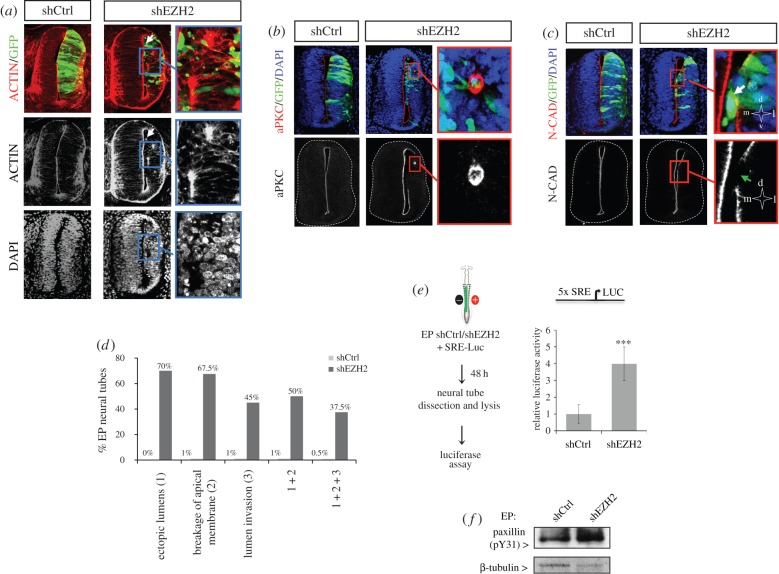
EZH2 preserves NE structure. (*a*–*c*) Transversal sections of neural tubes from HH11–12 embryos electroporated *in ovo* with shCtrl or shEZH2 and stained with phalloidin-rhodamine (*a*), aPKC (*b*) or N-cadherin (N-CAD, *c*) antibodies and DAPI 48 h PE. The results are representative of at least four independent experiments. White arrows mark progenitor cells that have lost the apicobasal polarity and have occupied the lumen. Green arrow indicates area where the apical membrane was severely disrupted along the luminal surface. (*d*) Graph shows the quantification of the morphological alterations observed in electroporated neural tubes. Data represent the percentage of *n* = 51 sections (from three to four embryos). (*e*) HH11–12 embryos were electroporated *in ovo* with shCtrl or shEZH2 together with pSRE-luc plasmid [24–26] and β-gal reporter used as internal control; 48 h PE the neural tubes were dissected out, the tissue was disaggregated and the luciferase activity measured using the Life Technologies dual kit. Data represent mean of three experiments from five to seven embryos. Error bars indicate s.d. ***p < 0.001. (*f*) HH11–12 embryos were electroporated *in ovo* with shCtrl or shEZH2; 48 h later GFP+ neural cells were FACS purified. Total protein extracts were prepared and the levels of paxillin (pY31) were determined by western blot. β-Tubulin levels were used to normalize the protein content.

Our data above demonstrate that shEZH2 electroporation altered NE morphology and organization. Considering that this is a totally new EZH2-related phenotype we further characterized the nature of the observed alterations. We immunostained electroporated neural tubes with phalloidin, which stains fibrillar actin. After shEZH2 electroporation, a subset of cells lost the apical junctions and invaded the neural tube lumen (figure 2*a*,*c* white arrows and *d*; also figure 1*c*, left panel) (percentage of EP neural tubes with lumen invasion: shCtrl 1%, shEZH2 45%). In addition, ectopic lumens were observed in the electroporated side of the neural tube (amplified in figure 2*a* and quantified in figure 2*d*) (percentage of EP neural tubes with ectopic lumens: shCtrl 0%, shEZH2 70%). Interestingly, the ectopic lumens were functional, as suggested by the mitotic divisions that took place at these new apical surfaces (electronic supplementary material, figure S3*c* and figure 1*d*, yellow arrows). These data suggest that the observed NE morphological alterations are related to defects in the apicobasal polarity. Interestingly, these effects were partially reverted after electroporation of rEZH2 (electronic supplementary material, figure S1*e*); however, no rescue was observed when using the catalytic mutant rEZH2DSET (electronic supplementary material figure S2*c*). To test this hypothesis, shEZH2 and shCtrl neural tubes were immunostained for aPKC, a basic component of the apical complex. Results in figure 2*b*,*d* and electronic supplementary material, figure S3*d* indicated that, after EZH2 depletion, the apical membrane was severely disrupted along the luminal surface (percentage of EP neural tubes with breakage of apical membrane: shCtrl 1%, shEZH2 67.5%), confirming the creation of new ectopic lumens (amplified in figure 2*b*). We know that, in addition to the apical complex, the integrity of the apical membrane requires a correct distribution of adherens junction (AJ) components. Therefore, we decided to analyse the distribution of N-cadherin after shEZH2 electroporation and found, after AJ protein immunostaining, that the apical membrane was disrupted (figure 2*c*, green arrow) and that some progenitors had lost the apicobasal polarity and had occupied the lumen (figure 2*c*, white arrow).

It has been proposed that EZH2 regulates actin polymerization by interacting with vav1, an activator of RhoA GTPase signalling [27], a pathway that couples developmental signals and downstream cytoskeletal rearrangements [28,29] and is repressed in the neural tubes at the analysed stages [30,31]. This gives us the rationale to explore the possibility that EZH2 depletion could affect Rho activity at the early stages of neurogenesis. To do this, we analysed Rho activity in EZH2-depleted or control embryos on the widely used serum response element (SRE) fused to luciferase gene reporter [24–26]. Figure 2*e* shows higher levels of luciferase activity in EZH2-depleted embryos, indicating an increase in Rho activity. Moreover, we observed higher levels of phosphorylated paxilin, a well-known Rho target [32], in shEZH2-electroporated embryos (figure 2*f*), suggesting that EZH2 depletion correlates with alteration of the Rho activity in the chick embryo neural tube.

### 2.3. EZH2 targets *p21^WAF1/CIP1^* promoter

**Figure 3.**
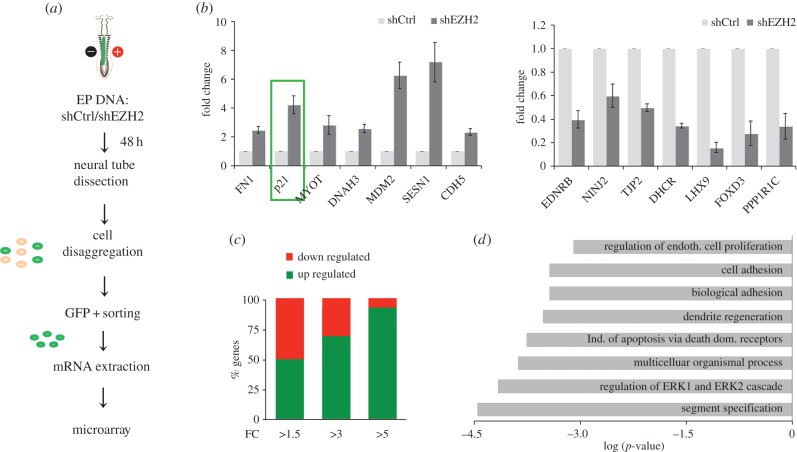
EZH2 regulates genes involved in proliferation and NE structure. (*a*) Schematic of microarray analysis design. (*b*) Relative mRNA levels were analysed for the indicated genes by qPCR in control (shCtrl) and EZH2-depleted cells (shEZH2) from electroporated neural tubes. Data represent mean of three independent experiments (five embryos for each replicate). Error bars indicate s.d. (*c*) Graph showing the percentage of upregulated and downregulated genes in the microarray experiment classified by FC. (*d*) Scheme showing the GO term enrichment of the genes deregulated (FC > 1.5) in EZH2-depleted cells.

To gain insight into the role of EZH2 in early neurogenesis, we analysed the transcriptional profiling of fluorescence-activated cell sorting (FACS)-purified GFP+ cells, from shEZH2 and shCtrl-electroporated neural tubes (figure 3*a*). We identified 462 genes that significantly changed their expression (fold change (FC) > 1.5 and *p*-value < 0.05) in shEZH2 samples (see Material and methods section). This result was validated by qPCR analysis of 14 randomly chosen genes (figure 3*b*). Interestingly, the proportion of upregulated and downregulated genes showing FC > 1.5 was similar. However, the genes with a higher FC were predominantly upregulated (figure 3*c*), in accordance with the repressor role described for EZH2. Gene ontology analysis revealed that genes misexpressed by EZH2 depletion were frequently associated with cell proliferation, cell adhesion and apoptosis regulatory processes, among others (figure 3*d*). Interestingly, we observed the increase of a Rho family member, *RhoBTB2*, as well as a decrease of the Rho inhibitory factor *ARHGAP10* that could contribute to the observed increased Rho activity. Moreover, changes in the expression of other genes involved in actin cytoskeleton and apicobasal polarity such as *SDK2*, *FZD3*, *FN1*, *NINJ1*, *NELL2* and *NINJ2* were identified. We also found that *p21^WAF1/CIP1^*, a well-known tumour suppressor gene and regulator of the Rho pathway, was highly upregulated (figure 3*b*).

**Figure 4.**
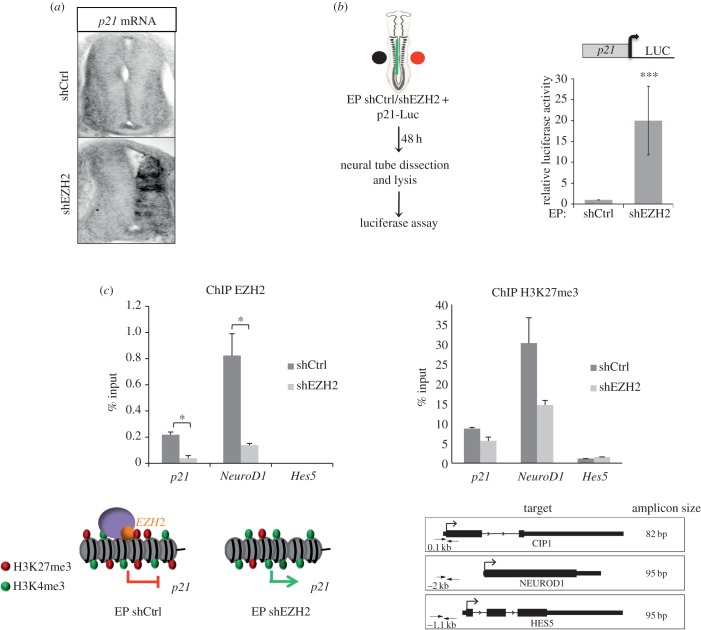
EZH2 targets *p21*^*WAF1/CIP1*^ promoter. (*a*) *p21^WAF1/CIP1^* ISH 48 h after *in ovo* electroporation of shCtrl or shEZH2 in HH11–12 embryo neural tubes. (*b*) HH11–12 embryos were electroporated *in ovo* with shCtrl or shEZH2 together with *p21^WAF1/CIP1^* promoter fused to the luciferase and pCMV-β-gal reporter used as internal control; 48 h PE the neural tubes were dissected, the tissue was disaggregated and the luciferase activity measured using the Life Technologies dual kit. Data represent mean of three experiments from four to six embryos. Error bars indicate s.d. ***p < 0.001. (*c*) ChIPs analysed by qPCR from HH11–12 electroporated neural tube cells with shCtrl or shEZH2 (48 h PE) using EZH2 or H3K27me3 antibodies at the *p21^WAF1/CIP1^*, *NeuroD1* and *Hes5* promoters. Results are represented as percentage of input. Map of the genes and the position and length of the amplicons are shown in the bottom part of the figure. Three biological replicates, with ten HH12 embryo neural tubes each, were used in each experiment. Error bars indicate s.d. *p < 0.05.

Given that p21^WAF1/CIP1^ expression is undetectable in the VZ of the neural tube at the analysed developmental stages (figure 4*a*), we postulated that EZH2 directly regulates *p21^WAF1/CIP1^* expression at this stage of development. We first confirmed its increase by *in situ* hybridization (ISH) of HH11–12 embryo transverse sections of EZH2-depleted embryos. Results showed that p21^WAF1/CIP1^ mRNA was highly induced after EZH2 depletion (figure 4*a*). Next, we analysed the activity of *p21^WAF1/CIP1^* promoter fused to a luciferase reporter in control and EZH2-depleted chick embryo neural tubes. As inferred by the results shown in figure 4*b*, EZH2 represses *p21^WAF1/CIP1^* promoter, either directly or indirectly, at the analysed developmental stages. Finally, we used chromatin immunoprecipitation (ChIP) experiments to test if this promoter was directly targeted by EZH2. In addition to *p21^WAF1/CIP1^*, we also analysed *NeuroD1* and *Hes5* promoters. *Hes5* was used as a negative control of ChIPs, because it is highly expressed in neural tubes [33] and, for this reason, unlikely to be a target of EZH2. *NeuroD1* was used as a positive control, as it is repressed at the analysed stages [33] and is a known H3K27me3 target in stem cells [22,34]. Figure 4*c* showed that *p21^WAF1/CIP1^* promoter was enriched in EZH2 at neural tubes. This enrichment was smaller than in the *NeuroD1* promoter; as expected, EZH2 was not detected at the *Hes5* promoter (figure 4*c*).

To examine whether changes in *p21^WAF1/CIP1^* expression were directly associated with EZH2 levels, we used ChIP assays at *p21^WAF1/CIP1^*, *NeuroD1* and *Hes5* promoters after *in ovo* electroporation with shCtrl or shEZH2. In figure 4*c*, left panel, we show that, after EZH2 depletion, EZH2 levels decreased at both *p21^WAF1/CIP1^* (5.25 ± 0.5-fold) and *NeuroD1* (5.9 ± 0.9-fold) promoters. These data suggest that *p21^WAF1/CIP1^* is directly repressed by EZH2 in the chick embryo neural tube, at the analysed developmental stages.

As EZH2 is the catalytic subunit of PRC2, we assessed if *p21^WAF1/CIP1^* promoter is trimethylated at H3K27 and if this mark changes upon EZH2 depletion, using ChIP assays. We found (figure 4*c*, right panel) that the *p21^WAF1/CIP1^* promoter was enriched in H3K27me3 at HH25 neural tubes. This enrichment was clearly smaller than in the *NeuroD1* promoter, but higher than in the *Hes5* promoter. Interestingly, this histone modification decreased (45% ± 0.1) upon EZH2 depletion and correlated with *p21^WAF1/CIP1^* promoter activation (figure 4*a*,*b*). These data suggest that EZH2-mediated H3K27me3 might regulate the expression of *p21^WAF1/CIP1^* gene at the analysed developmental stages.

### 2.4. EZH2-mediated repression of *p21*^*WAF1/CIP1*^ contributes to NE integrity

**Figure 5.**
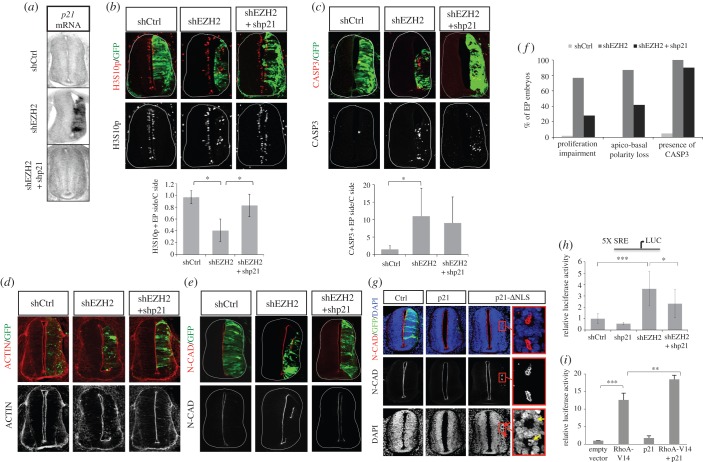
shp21 rescues neural progenitor proliferation and apicobasal polarity. (*a*) *p21^WAF1/CIP1^* ISH 48 h after *in ovo* electroporation of shCtrl, shEZH2 or shEZH2 and shp21 in HH11–12 embryo neural tubes. (*b*–*e*) Transversal sections of neural tube from HH11–12 embryos electroporated *in ovo* with shCtrl, shEZH2 or shEZH2 and shp21 and stained with H3S10p (*b*), caspase 3 (CASP3, *c*) and N-cadherin (N-CAD, *e*) antibodies or with phalloidin-rhodamine (*d*) 48 h PE. Graphs below the panels show the quantification of the corresponding immunostaining. Data represent mean of *n* = 20–40 sections (from four to six embryos). Error bars indicate s.d. *p < 0.05. (*f*) Graph showing the percentage of immunostained sections of Ctrl, shEZH2 and shEZH2 + shp21 that presents the indicated phenotypes. (*g*) Transversal sections of neural tubes from HH11–12 embryos electroporated *in ovo* with pCIG vector (Ctrl), pCIG together with *p21^WAF1/CIP1^* or cytoplasmic *p21^WAF1/CIP1^* (p21-ΔNLS) and stained with DAPI and N-cadherin 48 h PE. Ectopic lumens are amplified. Mitotic cells at the new lumens are visualized by DAPI staining (yellow arrows). The data are representative of at least three independent experiments. (*h*) HH11–12 embryos were electroporated *in ovo* with shCtrl, shp21, shEZH2 or shEZH2 and shp21 together with pSRE-luc plasmid and β-gal reporter used as internal control; 48 h later the neural tubes were dissected, the tissue was disaggregated and the luciferase activity measured using the Life Technologies dual kit. Data represent mean of three experiments from five to seven embryos. Error bars indicate s.d. *p < 0.05; ***p < 0.001. (*i*) 293 T cells were transfected with *RhoA-V14*, *p21^WAF1/CIP1^* or *RhoA-V14* and *p21^WAF1/CIP1^* expression vectors together with pSRE-luc plasmid and β-gal reporter used as internal control. 24 h later the luciferase activity measured using the Life Technologies dual kit. Data represent mean of four experiments. Error bars indicate s.d. **p < 0.01; ***p < 0.001.

Next, we analysed to what extent *p21^WAF1/CIP1^* derepression was responsible for the shEZH2-induced phenotype. To investigate if restoration of p21^WAF1/CIP1^ levels in shEZH2 embryos affects the final phenotype, we co-electroporated neural tubes with shEZH2 and an shRNA against *p21*^*WAF1/CIP1*^ (shp21), which efficiently reduced the p21^WAF1/CIP1^ levels (figure 5*a*). Then, we analysed BrdU incorporation and H3S10p signals by immunostaining assays. Interestingly, the proliferation defects observed by depletion of EZH2 were rescued upon co-electroporation of shp21 (figure 5*b*,*f*, electronic supplementary material, figure S4*a*) (percentage of BrdU+GFP+/total GFP+ cells: shCtrl 19.4 ± 6.1%, shEZH2 11.5 ± 4.9%; shEZH2 + shp21 22.6 ± 8.1%); percentage of H3S10p+ cells EP side/control side: shCtrl 0.97 ± 0.1%, shEZH2 0.41 ± 0.19%; shEZH2 + shp21 0.83 ± 0.19%).

Although the neural progenitors proliferated normally upon shEZH2 and shp21 co-electroporation, the caspase 3 levels remained high (difference of caspase 3+ cells EP side/control side: shCtrl 1 ± 0.7%, shEZH2 10.8 ± 8.8%; shEZH2 + shp21 9.5 ± 8.2%; figure 5*c*,*f*). This suggests that p21^WAF1/CIP1^ is not the main factor driving cell death after EZH2 depletion. Therefore, the survival phenotype induced by low levels of EZH2 was independent from the proliferation defects influenced by *p21^WAF1/CIP1^* misregulation. Consistent with this, data from expression microarrays identified some genes (*Fas*, *SesN1* and *ZBTB38*) directly involved in apoptosis that could be responsible for the observed phenotype.

We next examined if upregulation of *p21^WAF1/CIP1^* also contributes to the observed NE structural defects. With this aim, we analysed NE polarity with two apical and AJ markers (actin and N-cadherin). The data in figure 5*d*,*e*,*f* demonstrated that decreasing the p21^WAF1/CIP1^ levels partially (52%) rescued NE structural defects.

To discard proliferation and differentiation effects mediated by the nuclear p21^WAF1/CIP1^ in this regulation, we tested the phenotype resulting after electroporation of a *p21^WAF1/CIP1^* mutant. This mutant lacks the nuclear localization signal (NLS) (p21-ΔNLS) and localizes mainly in the cytoplasm (electronic supplementary material, figure S4*b*) [35]. We observed that electroporation of *p21^WAF1/CIP1^* induced cell cycle leave but not alteration of the NE structure. However, electroporation of cytoplasmic *p21^WAF1/CIP1^* (but not the nuclear *p21^WAF1/CIP1^*) in chick embryo neural tube induced the formation of ectopic and functional lumens. These were similar, but milder, to those observed in EZH2-depleted neural tubes (figure 5*g*, yellow arrows). Moreover, electroporation of another factor, Smad3 constitutively active, that induces neuronal differentiation at the analysed stages of development in the neural tube [36], did not affect the NE structure (electronic supplementary material, figure S4*c*), suggesting that p21^WAF1/CIP1^ by itself, and not indirect effects derived from its increase, contributes to NE integrity. These data proved that EZH2-mediated repression of *p21^WAF1/CIP1^* by itself is important for both the NE integrity and progenitor proliferation. Next, we tested whether p21^WAF1/CIP1^ could be involved in the observed Rho regulation in the neural tube. With this goal in mind, we measured the activity levels of SRE in shEZH2–shp21 co-electroporated embryos. In figure 5*h*, we show that depletion of p21^WAF1/CIP1^ partially rescued the increase on SRE activity induced by shEZH2 in the embryos. Additionally, overexpression of *p21^WAF1/CIP1^* strongly synergized with RhoA, to activate the SRE promoter in the 293 T cell line (figure 5*i*). These results suggest that the increase in Rho activity that correlates with EZH2 depletion was partly due to p21^WAF1/CIP1^.

Altogether, our results strongly suggest that EZH2 is essential to maintain neural tube homeostasis at early stages of vertebrate development.

## 3. Discussion

In this work, we demonstrate that EZH2 plays an essential role in vertebrate neurogenesis, using chick embryo spinal cord as a model. Our results revealed an unexpected role of EZH2 in controlling NE structure and integrity. We demonstrated that EZH2-depleted neural tubes lost the apicobasal polarity, and their morphological structure was altered. In addition, EZH2-depleted neural tubes were smaller than control neural tubes, due to defects in proliferation and an increase in cell death by apoptosis. Transcriptional and functional analysis confirmed that EZH2-mediated repression of *p21^WAF1/CIP1^* contributes to both NE integrity and proliferation.

### 3.1. Role of EZH2-mediated H3K27 methylation

Our results demonstrated that *p21^WAF1/CIP1^* expression induced by EZH2 depletion correlated with changes in H3K27 methylation at the promoter. Interestingly, we have not observed global changes on H3K27 methylation. A possible explanation of this result is that EZH1, whose expression increases after EZH2 depletion, partially replaces EZH2 at many but not all places in the genome, as has been proposed [37]. Thus, global H3K27me3 levels not always reflect the methylation/demethylation dynamics at a particular locus. Similar results have been shown for other chromatin regulators [38–40]. These results suggest that local changes in H3K27me3 at promoters drive the observed phenotype. In addition, the fact that EZH2DSET did not rescue it reinforces the idea that EZH2 catalytic activity on histone H3, or other substrates [41], is essential in early neurogenesis. Moreover, apart from its nuclear role, EZH2 cooperates with different signals to allow actin reorganization and proliferation [17]. All these published data suggest that EZH2 is a very versatile protein which could operate during early neurogenesis. Further characterization of the HMT-dependent and HMT-independent activities may reveal novel regulatory mechanisms of EZH2.

### 3.2. EZH2 and proliferation

Regarding the requirement of EZH2 for neural progenitor proliferation, our findings reveal notable differences in the observed mechanism by which PRC2 complex promotes proliferation in ESCc and cancer cells [12,14,20]. In this mechanism, EZH2 increases H3K27me3 levels in the Ink4/Arf locus, to inhibit the transcription of *p16^INK4A^* and *p19^ARF^*, which induces cell-cycle progression. We observed that the truncated chicken Ink4/Arf locus [42] was not affected by the depletion of EZH2 (data not shown). For this reason, we believe that this is an excellent model to identify new EZH2 targets involved in the regulation of neural progenitor proliferation. Our data revealed that EZH2-mediated regulation of a key inhibitor of the G1 to S transition, *p21^WAF1/CIP1^*, contributes to neural progenitor homeostasis. Interestingly, *p21^WAF1/CIP1^* has been shown to be regulated by Bmi-1, a member of the PRC1 complex, in neural stem cells to control cell renewal [43]. Considering that some promoters or genomic sites can recruit either one or/and the other PRC complex [44], it would be interesting to investigate whether Bmi-1 is directly involved in the repression of *p21^WAF1/CIP1^* promoter in the absence of EZH2. On the other hand, it could be possible that both complexes, PRC1 and PRC2, collaborate to regulate *p21^WAF1/CIP1^* promoter. In this case, it could be important to analyse the contribution of H3K27me3 and H2AK119 ubiquitination to this promoter activity. Our data strongly indicated that EZH2 directly targets *p21^WAF1/CIP1^* promoter in neural progenitors and that its activity correlates with changes in H3K27me3 levels. Whether PRC1 complex recognizes and binds to H3K27me3 at this promoter in neural progenitors is still an open question.

### 3.3. EZH2 and neural tube structure

Our results demonstrate that EZH2 depletion correlates with alteration of the Rho pathway. In agreement with this idea, it has been proposed that EZH2 regulates actin polymerization by interacting with vav1, an activator of RhoA GTPase signalling [27]. Moreover, EZH2 directly represses DLC1 (deleted in liver cancer 1), a GTPase-activating protein of the Rho family proteins, implicated in actin cytoskeleton reorganization [45]. More recently, it has been shown that EZH2 controls cofilin activity and consequently the actin cytoskeleton, regulating integrin alpha 2 expression in colon cancer cells [16].

On the other hand, there are several reports demonstrating the importance of Rho GTPases in the preservation of NE integrity in chick embryo neural tube [46]. In particular, a critical role of RhoA in the maintenance of apical AJs and the regulation of neural progenitor proliferation has been described in developing mammalian brain [47]. Interestingly, Rho accumulates apically in developing chick neural tube and is subjected to a strict spatio-temporal regulation [48]. In fact, both disruption and continuous activation of Rho activity altered neural tube formation and morphology [48]. One naturally wonders how EZH2 regulates Rho activity in the neural tube. We are still far from a complete understanding of this regulation; however, our data suggest that EZH2 contributes to maintaining proper Rho levels during neural tube development. Further experiments are required to totally clarify whether EZH2 directly represses Rho-related genes during neural development or/and non-transcriptional effects are also involved. Interestingly, inmunostaining experiments performed in our laboratory indicate that EZH2 has a cytoplasmic location at the ventricular zone of the neural tube (data not shown), suggesting a potential transcriptional-independent contribution of EZH2 to the observed phenotype.

### 3.4. EZH2 in development

Recently, it has been shown that senescence occurs during embryonic development at multiple locations, in a p21^WAF1/CIP1^-dependent manner, and independent of p53 and other cell-cycle regulators promoting tissue remodelling [49,50]. On the other hand, it has been proposed that EZH2 regulates senescence in different biological contexts such as cancer [20,51] or in primary cells [52]. These observations provided us with a rationale to propose that EZH2 could regulate senescence at early stages of neural development, through *p21^WAF1/CIP1^* repression. Accordingly, preliminary data from our laboratory show an intense senescence in the neural tube, mainly in the MZ where the EZH2 expression decreases. Therefore, EZH2-mediated *p21^WAF1/CIP1^* repression might be essential to maintain a proper NE structure and morphology at early stages of neurogenesis.

In summary, our findings contribute to clarify the function of EZH2 during the development of the nervous system, revealing a new function for this epigenetic regulator.

## 4. Material and Methods

### 4.1. Plasmids and recombinant proteins

DNA sequences coding two EZH2 and p21^WAF1/CIP1^ shRNAs were cloned into pSHIN vector [53] that contains the pSUPER shRNA expression cassette and another expression cassette for EGFP. Human EZH2 (rEZH2) and its deleted form lacking the SET domain (amino acids 622–707) cloned into pCIG vector [54] have been previously described [33]. *p21^WAF1/CIP1^*, *p21^WAF1/CIP1^* ΔNLS expression vectors and *p21^WAF1/CIP1^* promoter fused to luciferase reporter were a gift from Dr N. Agell [35]. pCIG-Smad3S/D was kindly provided by Dr E. Martí [36]. The 5XSRE fused to the luciferase-reporter gene was kindly given by Dr A. Aragay.

### 4.2. Primers for shRNAs

ChickEZH2(1)fw:

GATCCCCCAGACTCTCAATGCTGTTGCTTCAAGAGA GCAACAGCATTGAGAGTCTTTTTTGGAAA

ChickEZH2(1)rw:

AGCTTTTCCAAAAAAGACTCTCAATGCTGTTGCTCTCTTGAAGCAACAGCATTGAGAGTC TGGG

ChickEZH2(2)fw:

GATCCCCCCAACGTAGGATACAGCCTGTTCAAGAGAGTTGCATCCTATGTCGGACTTTTTGGAAA

ChickEZH2(2)rw:

AGCTTTTCCAAAAACAACGTAGGATACAGCCTGTCTCTTGAAGTTGCATCCTAT GTCGGACGGG

Chickp21^WAF1/CIP1^fw:

GATCCCCGCAGACCACCATCAAAGACTTCAAGAGACGTCTGGTGGTAGTTTCTGTTTTTGGAAA

Chickp21^WAF1/CIP1^rw: AGCTTTTCCAAAAAGCAGACCACCATCAAAGACTCTCTTGAACGTCTGGTGGTAGTTTCTGGGG

### 4.3. Antibodies

The following antibodies were used: anti-BrdU (Hybridoma Bank); anti-mouse β-tubulin III (Tuj1, Covance, MMS-435P); anti-H3S10p (Upstate); anti-caspase 3 (BD PharMingen); anti-N-cadherin (DSHB); anti-trimethyl H3K27 (Millipore); anti-EZH2 (Zymed and Active motif); anti-aPKC (Santa Cruz, sc-17781); anti-β-tubulin (Millipore, MAB3408); anti-SOX5 (kindly provided by Dr Morales [55]; anti-pY31 paxillin (Invitrogen), Actin was stained with phalloidin-rhodamine (Sigma, P1951).

### 4.4. Fluorescence-activated cell sorting

Electroporated embryos were dissected out 48 h PE and trypsinized for 5–10 min in 0.5% trypsin-EDTA (Sigma). Trypsinization was stopped by 20% horse serum in PBS −0.1% glucose solution. GFP+ cells from the suspension were sorted by flow cytometry using a MoFlo flow cytometer (DakoCytomation, Fort Collins, CO, USA).

### 4.5. Chick *in ovo* electroporation

Eggs from White-Leghorn chickens were incubated at 38.5°C and 70% humidity. Embryos were staged following HH [56]. Embryos were electroporated with purified plasmid DNA at 0.25–3 µg µl^−1^ in H_2_O with 50 ng ml^−1^ of fast Green. Plasmid DNA was injected into the lumen of embryos, electrodes were placed at both sides of the neural tube and embryos were electroporated by an IntracelDual Pulse (TSS-100) electroporator delivering five 50 ms square pulses of 20–25 V. Electroporated embryos were allowed to develop for 24 h or 48 h and then dissected out and processed for the appropriate protocol.

### 4.6. Indirect immunofluorescence

The brachial regions of the embryos were collected and fixed for 2 h at 4°C in 4% paraformaldehyde. They were then rinsed, embedded in 5% agarose–10% sacarose for sectioning in a Leica vibratome, or immersed in PBS 30% sucrose solution and embedded in OCT for sectioning in a Leica cryostat (CM 1900). Sections were blocked at room temperature for 1 h in 1% bovine serum albumin (in PBS with 0.1% Triton-X) before overnight incubation at 4°C with primary antibodies. Finally, sections were incubated for 2 h at room temperature with Alexa-conjugated goat secondary IgG antibodies (Jackson Immuno Research Inc.) and 0.1 ng µl^−1^ DAPI (Sigma). Images were captured by a Leica SP5 confocal microscope using LAS-AF software.

### 4.7. *In situ* hybridization

Whole-mount embryo RNA ISH was done following standard procedures using EST bank probes for chick *p21^WAF1/CIP1^*. After hybridization, embryos were post-fixed in 4% paraformaldehyde for 2 h, embedded in 10% sucrose, 5% agarose solution and sectioned in a Leica vibratome (VT 1000S).

### 4.8. mRNA extraction and quantitative PCR

mRNA from HH10 and HH30 embryo neural tubes or from FACS-separated cells from dissected shRNA-control or shRNA-EZH2 electroporated neural tubes was extracted by TRIZOL (Invitrogen) protocol with 2 µl of pellet paint co-precipitant (Novagene). Reverse transcription was performed with Transcriptor kit (Roche), following the manufacturer's procedure. qPCR was performed with Sybergreen (Roche) in an LC480 Lightcycler (Roche). GAPDH was used for normalization.

### 4.9. Chromatin immunoprecipitation

shRNA-control or shRNA-EZH2 electroporated neural tubes were dissected out from HH25 embryos. 2.5 and 1.5 million cells were used for EZH2 ChIP and H3K27me3 ChIP, respectively, which were run essentially as described elsewhere [57]. Basically, cells were fixed with 1% formaldehyde for 10 min at room temperature; in addition, for EZH2 ChIP a previous fixation step with 2 mM DSG (Sigma 80424) was performed for 40 min at room temperature. Fixation was quenched with glycine (0.125 M). Fixed cells were lysed in a SDS-containing buffer (1% SDS; 10 mM EDTA pH 8.0; 50 mM Tris–HCl pH 8.1), and sonication was carried out in a Bioruptor (six cycles, high power, 30 s ON–30 s OFF) to get DNA fragments of an average of 500 bp. Chromatin was purified by centrifugation for 30 min, at 20 000*g* and 4°C. Purified chromatin was further diluted in immunoprecipitation buffer (0.01% SDS, 1.1% Triton X-100; 1.2 mM EDTA pH 8.0; 16.7 mM Tris–HCl pH 8.1; 167 mM NaCl). Incubation with the appropriated specific antibodies (EZH2 (AC22), Active Motif and H3K27me3 (07–449), Millipore) or an unspecific antibody (rabbit IgGs (C15410206), Diagenode) was performed overnight at 4°C. Protein A-bound beads were added and immunocomplexes were washed once with buffers TSE I (0.1% SDS; 1% Triton-X100; 2 mM EDTA pH 8.0; 20 mM Tris–HCl pH 8.1; 150 mM NaCl), TSE II (0.1% SDS; 1% Triton-X100; 2 mM EDTA pH 8.0; 20 mM Tris–HCl pH 8.1; 500 mM NaCl) and TSE III (0.25 M LiCl; 1% NP-40; 1% sodium deoxycholate; 1 mM EDTA pH 8.0; 10 mM Tris–HCl pH 8.1) and twice with TE buffer (10 mM Tris–HCl pH 8.1 and 1 mM EDTA). De-crosslinking was carried out at 65°C in elution buffer (1% SDS, 0.1 M NaHCO_3_) for 4 h. DNA was purified by phenol–chloroform followed by ethanol precipitation. Binding of the proteins of interest to the selected regions was assayed by qPCR in a Lightcycler 480 with SYBR Green (Roche) with the following primers:

fwp21^WAF1/CIP1^ GCAGATCCAGAACGACTTTGA; rwp p21^WAF1/CIP1^ GGTCTCGAAGTTGAAGTTCCA; fwHes5 TGAAAGATTGGCAGAGGAAAC; rwHes5 GTACCCATTTCTCACTACAGC; fwNeuroD1 GAGGGATTTTAACCACCTTCG; rwNeuroD1 TCACTTAGCGCGTGATTTACA.

### 4.10. *In vivo* luciferase-reporter assay

Chick embryos were electroporated at HH11–12 with the indicated DNAs together with the *p21^WAF1/CIP1^* promoter fused to luciferase reporter construct or the reporter plasmid pSRE-luc containing 5XSRE of the c-fos promoter and a β-gal-reporter carrying the CMV immediate early enhancer promoter for normalization. Embryos were harvested after 48 h PE and GFP-positive neural tubes were dissected and homogenized in Passive Lysis Buffer. Firefly- and β-gal-luciferase activities were measured by the β-gal and luciferase dual reporter assay (Dual-Light^®^ Luciferase and β-Galactosidase Reporter Gene Assay System, Life Technologies).

### 4.11. BrdU incorporation

A total 0.5 µg ml^−1^ BrdU was injected into the lumen of the neural tube of chick embryos 30 min before fixation. BrdU was detected on sections by treatment with HCl 2 N for 30 min, neutralization with sodium borate 0.1 M (pH 8.5) and incubation with anti-BrdU antibody.

### 4.12. Microarray analysis

Fifteen embryo neural tubes were electroporated with shRNA-control (shCtrl) or shRNA-EZH2 (shEZH2) for each of the three replicates. Isolated neural tubes were trypsinized and 100 000 GFP-positive cells were FACS purified for each replicate. RNA was extracted and after quality control, quantification, reverse transcription and labelling, it was independently hybridized to Affymetrix Chicken GeneChip using Affymetrix technology following manufacturer's instructions at IRB Barcelona's Functional Genomics Core Facility. Data analysis was performed using the GeneSpring software 10.0 (Silicon Genetics, Redwood City, CA, USA). Raw data from the six hybridizations were normalized to the 50th percentile per chip and to the median per gene. Normalized mean values for the two individual experimental groups (shCtrl and shEZH2) were generated for the experimental interpretation. The statistical significance of the differences was evaluated by using unpaired *t*-test. Differentially expressed genes were identified when the absolute FC > 1.5 and *p* < 0.05.

### 4.13. Primers used for qPCR

fwEZH2 CTCAATGTTTCCAGATAAGGGT; rwEZH2 GCTGTTCAGTGAGTTCTTTG; fwGAPDH CGATCTGAACTACATGGTTTAC; rwGAPDH ATCACAAGTTTCCCGTTCTC; fwp21^WAF1/CIP1^ CAGACCACCATCAAAGACTTCTA; rwp21^WAF1/CIP1^ CTTGGGCTTATCGTGGACAA; fwDNAH3 AATACACCATCCCCATTGAC; rwDNAH3 ATTGTGTTCTCCTGCTTCAT; fwMYOT GCAACACATACAAACAAGCC; rwMYOT CTTGCTGAAAGTTGGTCGGA; fwFN CCAGTTCACACAGAGATACC; rwFN GTGTGTCTGCCTGTACATCTA;

fwDHCR GAAAAGTTTGTGAGAAGCGTG; rwDHCR GGTCATGTAGCAATCCGCATA; fwTJP2 GAGATGGATCATAAGGCAAGAC; rwTJP2 GATGCTTCTGGGCTATTTCAA; fwNINJ2 CAGGATCTGAACAGCACAAC; rwNINJ2 TCTGGATTCTTTGCTGGGAT; fwEDNRB GTAATGGACTACATTGGCATTAAC rwEDNRB TTTGGAATCTCTTGCTCACC;fwMDM2 AGGAGGACTTCAGGGAAC; rwMDM2 GCTAACAGGCAGACTGG; fwSESN1 GAGAAGGAGACCGTTCAAG; rwSESN1 AAGAGTCCGCAAACAGC; fwCDH5 TTTCCCACACCTGACGTTAC; rwCDH5 TGTCCTAAGAGACCCACTTC; fwFOXD3 GCGAGTTCATCAGCAACC; rwFOXD3 GATGCTGTTCTGCCAGG; fwPPP1R1C AGGAGGAAGAGGGAATCG; rwPPP1R1C AGACTTGCATGGTTTAGTGG; fwLHX9 GAGAACGAGGCAGATCAC; rwLHX9 CGCTTTGTCTTCTGGGATG; fwPARD3 CCAGGTGAAGGGTTCCAGAC; rwPARD3 CCCCCATGTAGCCGTTTCTT; fwaPKC GAGCTGTATGGGAAGCGAGG; rwaPKC CCGCGGCCTGAAGTTGTTTA; fwCDH2A GCCCACGGAGTTTGTAGTG; rwCDH2A TGCTGGTTCAGGGGTTAGC; fwEZH1 TTGCCAACCACTCCGTGAA; rwEZH1 TCACCATCACAACTTTGGCATAG.

### 4.14. Statistical analysis

Quantitative data were expressed as mean and standard deviation. Significant differences between groups were tested by Student's *t*-test.

## Supplementary Material

Supplementary Figures and Figure Legends

## Data accessibility

Normalized data are available at the Gene Expression Omnibus database (http://www.ncbi.nlm.nih.gov/geo/query/acc.cgi?token=tvgtngkuuswwyju&acc=GSE39450) with the accession number GSE39450.
