# Supplementary material for: EZH2 regulates neuroepithelium structure and neuroblast proliferation by repressing p21
Source: Open Biol. 2016 Apr 20;6(4):150227. doi: 10.1098/rsob.150227 (PMC4852452; doi:10.1098/rsob.150227)

ELECTRONIC SUPPLEMENTARY MATERIAL

This file contains the Figures and Figure legends corresponding to the Supplementary Figures 1, 2, 3 and 4 of the manuscript:

EZH2 regulates neuroepithelium structure and neuroblast proliferation by repressing p21

Naiara Akizu*, Alejandra García*, Conchi Estarás, Raquel Fueyo, Carmen Badosa, Xavier de la Cruz and Marian A. Martínez-Balbás

SUPPLEMENTARY FIGURE LEGENDS

Supplementary Figure S1

(a) HH11-12 embryos were electroporated with shEZH2(1) or shEZH2(2) or shRNA-control (shCtrl) cloned into pSHIN vector expressing GFP. 48-PE neural tubes were dissected out. GFP+ cells were sorted by FACS and mRNA was extracted and purified. The same amount of total mRNA was retro-transcribed for qPCR analysis and the EZH2 relative mRNA was normalised by GAPDH. Results are mean of two independent experiments. Error bars indicate s.d. ***$p<0.001$.

(b) Transversal sections of neural tubes from HH11-12 embryos electroporated *in ovo* with shCtrl or shEZH2(1) or shEZH2(2) cloned into pSHIN vector expressing GFP and stained with DAPI 48h-PE. The distances from the central line of the lumen to the dorsal, medial and ventral ends of the neural tubes are marked.

(c) HH11-12 embryos were electroporated with a mix of shEZH2(1) and shEZH2(2) (that we named shEZH2) or shRNA-control (shCtrl). 48-PE neural tubes were dissected out. GFP+ cells were sorted by FACS. The mRNA was extracted and the EZH2 mRNA was quantified by qPCR

and normalised by GAPDH. Results are mean of two independent experiments. Error bars indicate s.d. * $p<0.05$.

(d) HH11-12 embryos were electroporated with shEZH2 or shCtrl cloned into pSHIN vector expressing GFP. 48h-PE neural tubes were dissected out. Disaggregated cells were stained in suspension for EZH2 and DAPI (righ panel) or used in an immunoblot with the indicated antibodies (left panel). Figure is representative of at least two biological independent experiments. Graph shows the quantification of EZH2 relative to b-tubulin signals in the immunoblots from either shCtrl or shEZH2-EP neural tubes. Error bars indicate s.d.

(e) Transversal sections of neural tubes from HH11-12 embryos electroporated *in ovo* with shCtrl or shEZH2 or shEZH2 together with a resistant EZH2 (rEZH2) stained with DAPI and with phalloidin, which stains fibrillar actin 48h-PE. The distances from the central line of the lumen to the dorsal, medial and ventral ends of the neural tubes are marked. Graph shows the quantification of the EP neural tubes that have reduced size and structurally altered electroporated side. Data represent mean of n=8.

Supplementary Figure S2

(a) Transversal sections of neural tube from HH11-12 embryos electroporated *in ovo* with shEZH2 cloned into pSHIN vector expressing GFP and stained with anti H3K27me3 48h-PE. GFP+ cells expressing the shRNA are indicated with red arrows. Graph shows the quantification of the H3K27me3 after shEZH2 EP in GFP+ (expressing shEZH2) and GFP- cells. Data represent the mean of n=20 to 30 sections (from 3 to 4 embryos).

(b) HH11-12 embryos were electroporated with shCtrl or shEZH2. 48h-PE neural tubes were dissected out. GFP+ cells were sorted by FACS and mRNA was extracted and purified. The same amount of total mRNA was retro-transcribed for qPCR analysis and the EZH1 relative mRNA

was normalised by GAPDH. Results are mean of two independent experiments. Error bars indicate s.d. * $p<0.05$.

(c) Transversal sections of neural tubes from HH11-12 embryos electroporated *in ovo* with shCtrl or shEZH2 or shEZH2 together with a rEZH2 or rEZH2DSET stained with DAPI and with phalloidin, which stains fibrillar actin 48h-PE. The distances from the central line of the lumen to the dorsal, medial and ventral ends of the neural tubes are marked. Graph shows the quantification of the EP neural tubes that have reduced size and structurally altered electroporated side. Data represent mean of n=8.

Supplementary Figure S3

(a-c) Transversal sections of neural tube from HH11-112 embryos electroporated *in ovo* with shCtrl or shEZH2 and stained with BrdU (a), Tuj1 (b) antibodies or with phalloidin-rhodamine and DAPI (c). Graphs on each panel show the quantification (a) or distribution (b) of the immunostaining. Data represent the mean of n=20 to 30 sections (from 4 to 6 embryos). Error bars indicate s.d. **$p<0.01$.

(d) Transversal sections of neural tube from HH11-112 embryos electroporated *in ovo* with shCtrl or shEZH2(1) or shEZH2(2) and stained aPKC and DAPI 48h-PE. The results are representative of at least 3 independent experiments. Green arrows and bar indicated areas where the apical membrane was severely disrupted along the luminal surface.

Supplementary Figure S4

(a) Transversal sections of neural tube from HH11-12 embryos electroporated *in ovo* with shCtrl, shEZH2 or shEZH2 and shp21 and stained with BrdU 48h-PE. Graphs below the panels show the quantification of the corresponding immunostaining. Data represent mean of n=20 to 40 sections (from 4 to 6 embryos). Error bars indicate s.d. * $p<0.05$; **$p<0.01$.

(b) Transversal sections of neural tube from HH11-12 embryos electroporated *in ovo* with *p21*$^{WAF1/CIP1}$ or *p21*$^{WAF1/CIP1}$ mutant that lacks the nuclear localization signal (p21-ΔNLS) fused to GFP (Rodriguez-Vilarrupla et al., 2002) stained with DAPI 48h-PE.

(c) Transversal sections of neural tubes from HH11-12 embryos electroporated *in ovo* with pCIG vector (Ctrl), or Smad3 constitutively active (Smad3 S/D) and stained with Tuj antibody, phalloidin-rhodamine and DAPI 48h-PE. The results are representative of at least 3 biologically independent experiments.

REFERENCES

Rodriguez-Vilarrupla, A., Diaz, C., Canela, N., Rahn, H. P., Bachs, O. and Agell, N. (2002). Identification of the nuclear localization signal of p21(cip1) and consequences of its mutation on cell proliferation. *FEBS Lett* **531**, 319-323.

Supplementary Figure S1

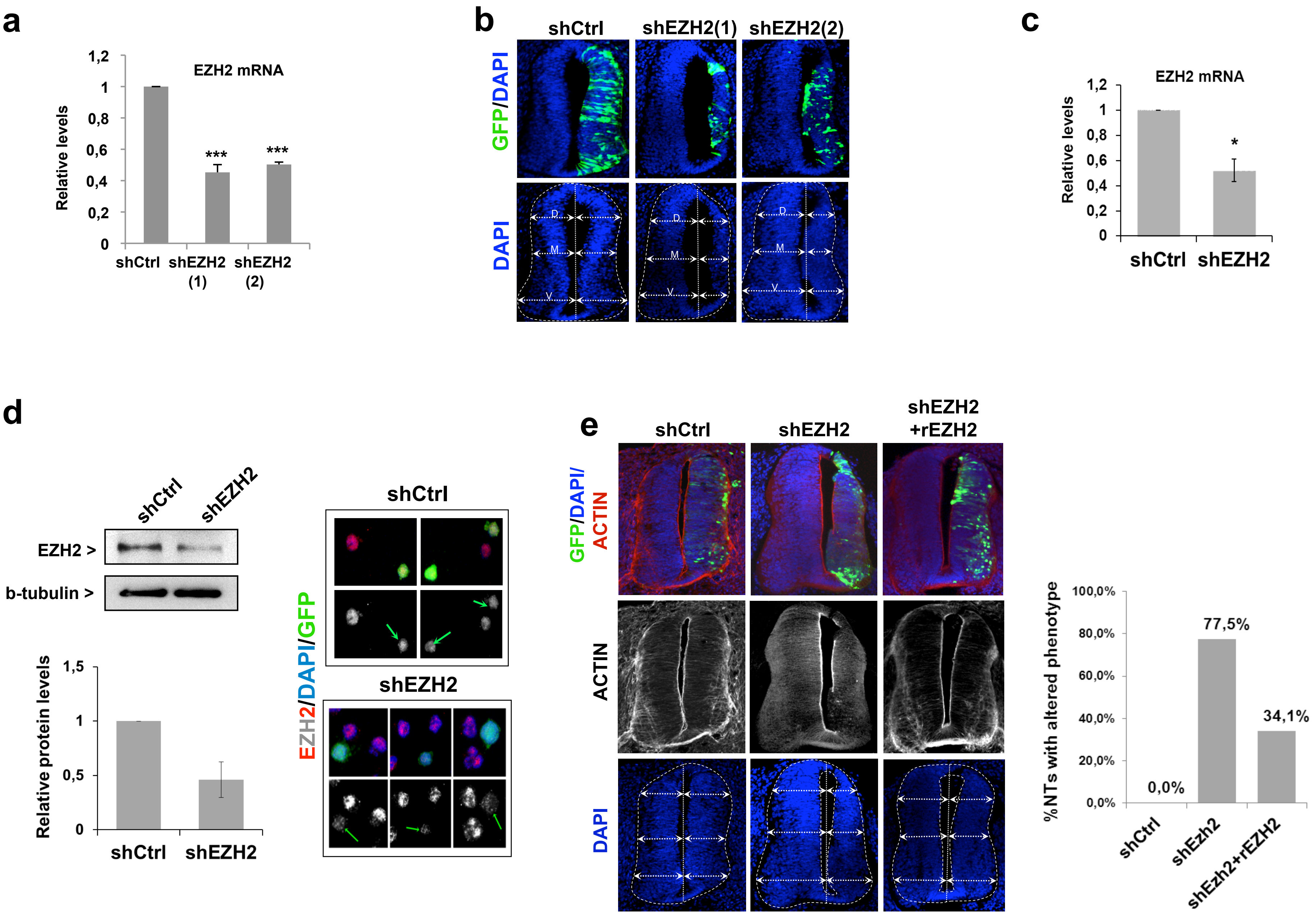

Supplementary Figure S2

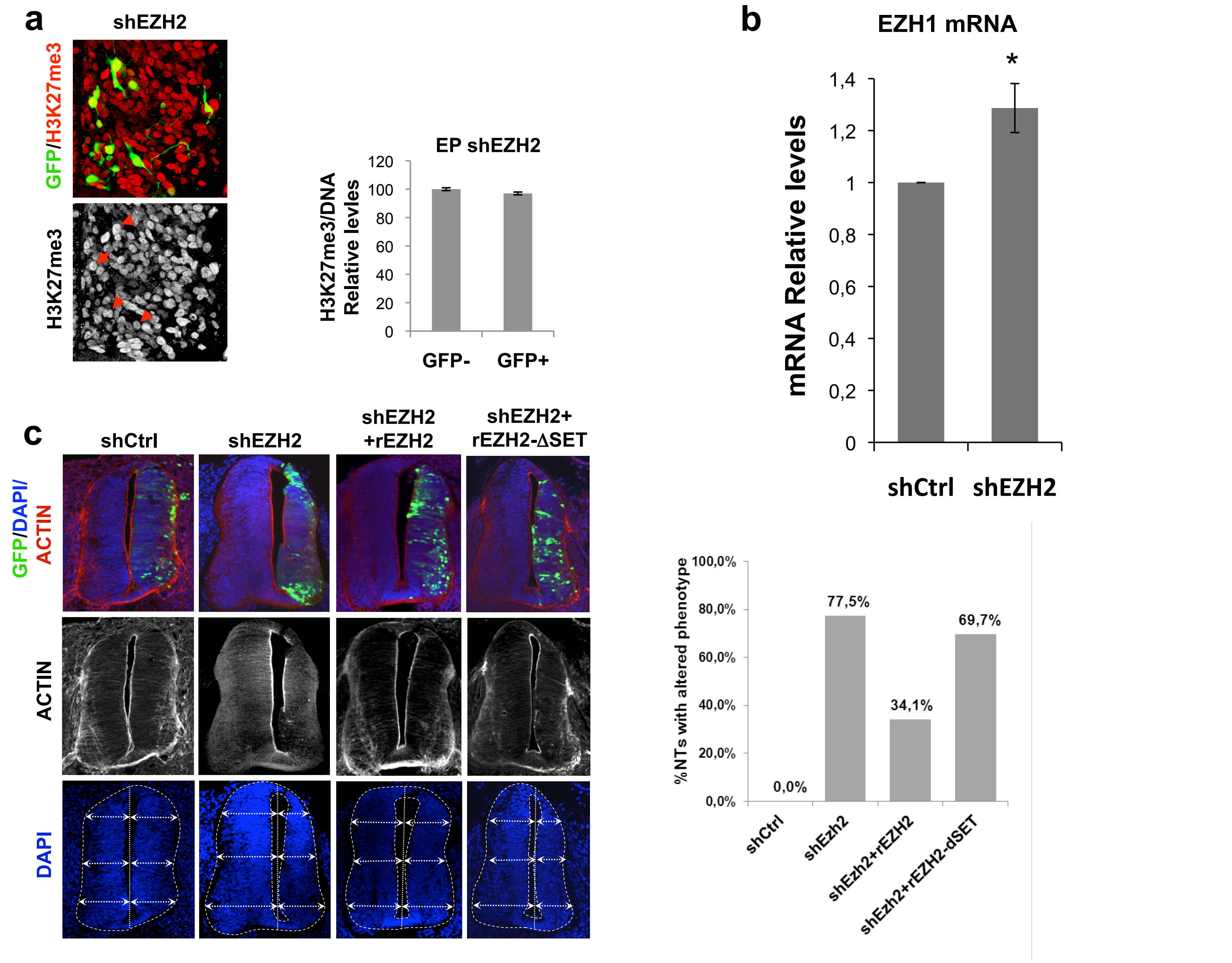

Supplementary Figure S3

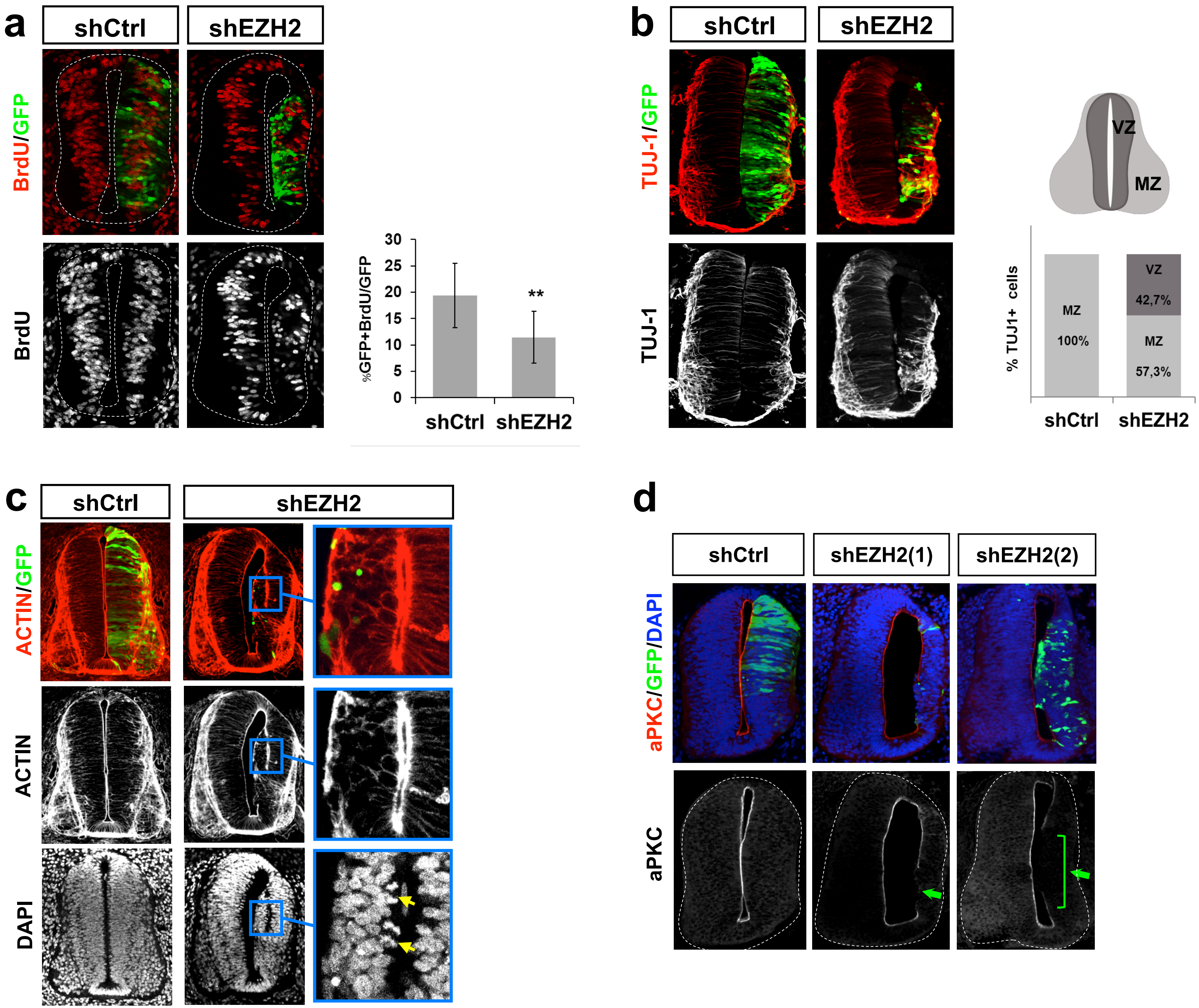

Supplementary Figure S4

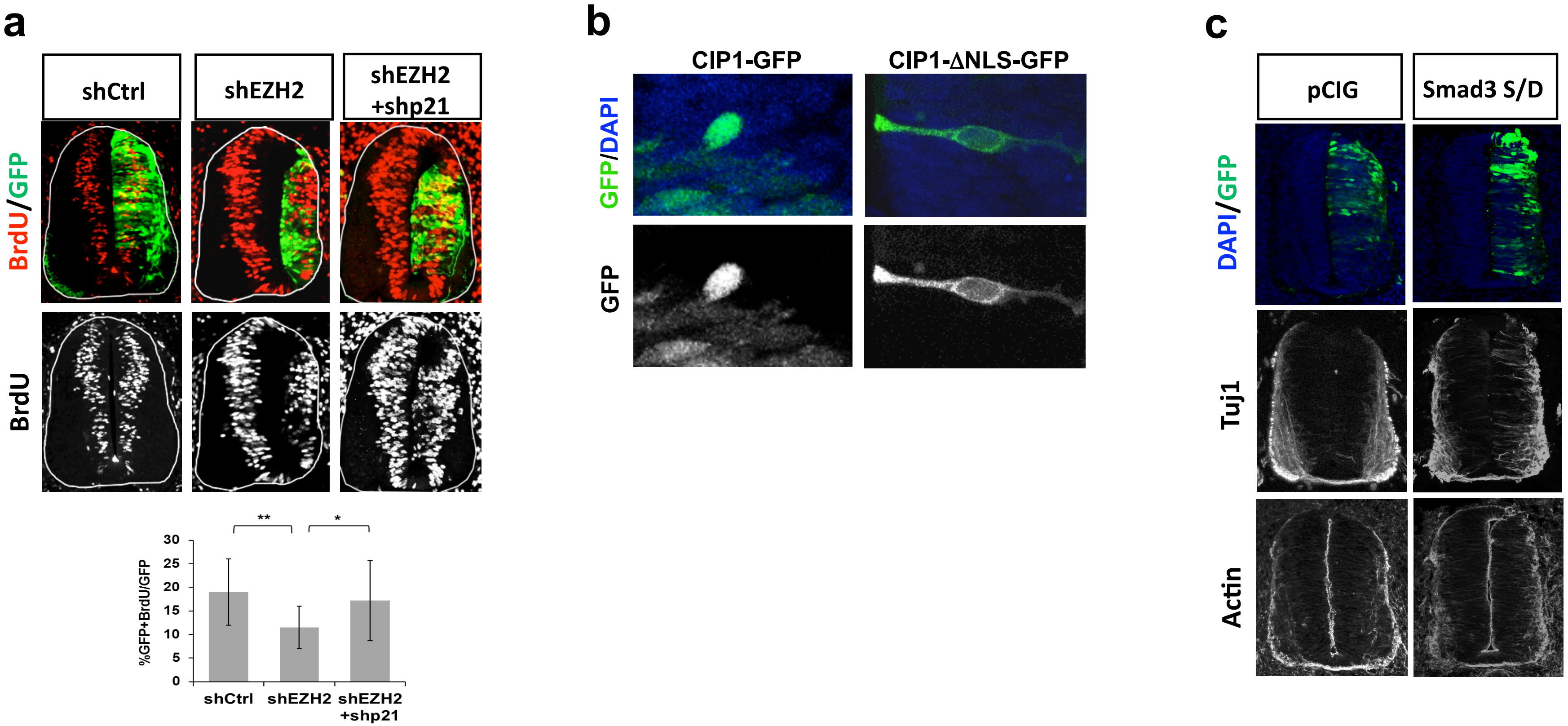

Supplement: Supplementary Figures and Figure Legends [file rsob150227supp1.pdf]
